# An updated, comprehensive meta-analysis of the treatment of anti-NMDAR encephalitis: Analysis, equipoise, and the urgent need for evidence over anecdote

**DOI:** 10.1016/j.jneuroim.2025.578651

**Published:** 2025-05-27

**Authors:** Yoji Hoshina, Tammy L. Smith, Alen Delic, Ka-Ho Wong, Lisa K. Peterson, Anastasia Zekeridou, Albert Aboseif, Christopher Coffey, Melissa A. Wright, Brenda Banwell, Annalisa Dialino-Felix, Susan Flavin, Lisa Dill, Maarten J. Titulaer, Gregory S. Day, Stacey L. Clardy

**Affiliations:** a Department of Neurology, University of Utah, Salt Lake City, UT, United States of America; b Department of Veterans Affairs Geriatric Research Education and Clinical Center, Salt Lake City, UT, United States of America; c George E. Wahlen Veterans Affairs Medical Center, Salt Lake City, UT, United States of America; d ARUP Institute for Clinical and Experimental Pathology, Salt Lake City, UT, United States of America; e Department of Pathology, University of Utah, Salt Lake City, UT, United States of America; f Department of Neurology, Laboratory Medicine and Pathology and Center for MS and Autoimmune Neurology, Mayo Clinic Minnesota, Rochester, MN, United States of America; g Department of Biostatistics, University of Iowa, Iowa City, IA, United States of America; h Division of Pediatric Neurology, Department of Pediatrics, University of Utah, Salt Lake City, UT, United States of America; i Department of Pediatrics, Johns Hopkins University, Baltimore, MD, United States of America; j Amgen Inc., Thousand Oaks, CA, United States of America; k Department of Neurology, Erasmus University Medical Center, Rotterdam, the Netherlands; l Department of Neurology, Mayo Clinic Florida, Jacksonville, FL, United States of America

**Keywords:** Anti-*N*-methyl-d-aspartate receptor, encephalitis, Autoimmune encephalitis, Immunotherapy

## Abstract

**Background:**

There are no FDA-approved treatments for anti-*N*-methyl-d-aspartate receptor encephalitis (NMDARE), and no prospective, multicenter clinical trials have been completed to provide evidence for management of this disease. We evaluated changes in treatment strategies and outcomes since the previous comprehensive review in 2019.

**Methods:**

A systematic literature review was conducted in PubMed capturing manuscripts published from January 2019 through March 2024. Demographic and clinical data were extracted from articles containing individual immunotherapy data in NMDARE and were compared to a previously published literature review. Studies with ≥10 cases, ≥6 months follow-up, and outcomes reported as favorable (modified Rankin Scale [mRS] 0–2) and poor (mRS 3–5 or 3–6), were grouped and summarized by diagnosis period.

**Results:**

Among 649 patients from 321 articles, first- (from 91.3 % to 98.3 %, *p* < 0.001) and second-line (from 31.8 % to 42.5 %, p < 0.001) immunotherapy use has increased. The proportion of patients receiving immunotherapy within 30 days of symptom onset increased from 50.1 % to 72.5 % (p < 0.001). Favorable outcome (mRS 0–2) increased from 71.5 % to 76.7 % (*p* = 0.024), while mortality (6.3 % to 6.9 %, *p* = 0.714) and relapse rates (12.6 % vs. 13.2 %, *p* = 0.789) remained unchanged. Data from larger cohort studies of patients diagnosed with NMDARE after 2013 have reported wide variability in the proportion of patients achieving a favorable outcome at final follow-up, ranging from 56 % to 93 %, depending on the institution and follow-up duration.

**Conclusions:**

Since 2019, more patients have been treated early with first- and second-line immunotherapies. Functional outcomes have shown modest improvements, whereas mortality and relapse rates have remained unchanged.

## 1. Introduction

Autoimmune encephalitis resulting from antibodies to central nervous system (CNS) *N*-methyl-d-aspartate receptors (NMDAR) was first defined in 2007 (Dalmau et al., 2007). Since its recognition, progress has been made in increasing awareness of this rare disease and in understanding the biological mechanisms that drive anti-NMDAR encephalitis (NMDARE) (Dalmau et al., 2019). Despite these advances, no high-quality, prospective, multicenter clinical trials have been completed to inform treatment decisions in NMDARE.

One important barrier to clinical trial enrollment is establishing clinical equipoise — a state of genuine uncertainty about the superiority of one treatment arm over another (AlHamaly et al., 2023). In rare diseases, such as NMDARE, equipoise may be obscured by undue weight assigned to treatment recommendations based on expert opinion (Abboud et al., 2021a; Abboud et al., 2021b; Nosadini et al., 2021a). Although helpful in framing an approach to a novel disease, documents that present “proposed best practices” or “international consensus guidelines” may unintentionally obfuscate the need for higher quality studies that are required to optimize diagnosis, treatment, and outcomes in patients with rare diseases. When broadly accepted, such recommendations often become “standard of care”, without the rigor of unbiased clinical trials. This acceptance can in turn raise concerns about clinical equipoise that may paradoxically limit advances in the field by challenging enrollment in clinical trials designed to evaluate alternate approaches to patient management.

In NMDARE, a landmark retrospective cohort analysis published in 2013 emphasized the association between early initiation of first-line immunotherapies (corticosteroids, intravenous immune globulin [IVIg], and plasma exchange) and better outcomes, as measured by modified Rankin Scale (mRS), as well as the utility of second-line immunotherapies (rituximab, cyclophosphamide) in treatment-refractory patients (Titulaer et al., 2013). These data informed subsequent consensus recommendations, with rapid adaptation and application in adult and pediatric populations (Abboud et al., 2021a; Abboud et al., 2021b; Nosadini et al., 2021a; Graus et al., 2016). A systematic literature review conducted in 2019 comparing publications from before 2013 and between 2013 and 2019 found that, although there was a sustained rise in the proportion of patients with NMDARE receiving first- and second-line immunotherapies, and the proportion of hospitalizations lasting ≥60 days decreased, patient functional outcomes remained unchanged (Nosadini et al., 2021b). The disconnect between evolving practice patterns and static outcomes highlights clinical equipoise in NMDARE and frames the need for high-quality trials to inform future treatments.

Much has changed since the last comprehensive literature review of treatment and outcomes in NMDARE was published in 2019, including the initiation of two randomized controlled clinical trials (i.e., ExTINGUISH trial [NCT04372615] and CIELO [NCT05503264]). As such, we conducted an updated systematic literature review to determine whether changes in this nascent field have resulted in changes in treatment strategies and outcomes in patients with NMDARE.

## 2. Methods

### 2.1. Systematic literature review for individual patient data

We conducted a systematic literature review in PubMed spanning manuscripts published from January 1, 2019 to March 31, 2024 using the following search terms: (anti–*N*-methyl-d-aspartate receptor encephalitis [Title/Abstract]) OR (*N*-methyl-d-aspartate antibody encephalitis[Title/Abstract]) OR (anti-NMDAR encephalitis[Title/Abstract]) OR (anti-NMDA receptor encephalitis[Title/Abstract]) OR (NMDA receptor encephalitis[Title/Abstract]) OR (anti-*N*-methyl- D-aspartate receptor antibody encephalitis[Title/Abstract]) AND (English [Language]) AND (“2019/01/01”[Date - Publication]: “2024/03/31”[Date - Publication]) NOT (review [Publication Type]). The database search was performed manually. Titles and abstracts were screened independently for relevance by two authors (Y.H. and T.S.). Publications were included if they met the following criteria: (1) patients with NMDARE with positive NMDAR antibodies in serum and/or cerebrospinal fluid (CSF) and (2) studies providing individual patient data on immunotherapy. Publications were excluded if they met any of the following criteria: (1) negative NMDAR antibodies in CSF, (2) NMDARE preceded by CNS infection (e.g., herpes simplex virus encephalitis), (3) NMDARE with coexisting CNS inflammatory disorders (e.g., myelin oligodendrocyte glycoprotein antibody disease), and (4) duplicated cases from previous reports. Neurologic severity in the acute phase and functional outcomes at the last follow-up were assessed via the mRS score. If not reported, mRS scores were retrospectively assigned based on clinical data. Favorable outcomes were defined as mRS 0–2 and poor outcomes as mRS 3–5 or mRS 6. Case reports and case series containing individual immunotherapy data published between 2019 and 2024 (the present study) were compared with data from a 2019 systematic literature review, which included clinical and immunotherapy data from publications prior to 2019 (Nosadini et al., 2021b). We also compared the post-2019 data (the present study) to the 2013–2019 subset published in the same prior literature review (Nosadini et al., 2021b), in order to facilitate comparison only with data from a more proximate time period. To assess the applicability of the included cases to the general NMDARE population, we categorized each study based on the primary feature it emphasized. This approach was motivated by concerns that recent publications may disproportionately focus on atypical, relapsing, or otherwise exceptional cases. Studies were grouped into one of the following categories: (1) relapsing or treatment-refractory disease course; (2) unusual neoplasm association; (3) emerging or experimental treatment; (4) emerging diagnostic technology; (5) unusual demographic or radiological findings; (6) unusual clinical presentation; and (7) relatively typical presentation. This study adhered to the Preferred Reporting Items for Systematic Reviews and Meta-analyses (PRISMA) guidelines.

### 2.2. Summarization of larger cohort data

Since restricting our analysis to publications with individual patient data on immunotherapy identified primarily case reports and small case series, a second part of the study focusing on larger cohorts was conducted. For this part, we searched PubMed for publications within the same timeframe (January 1, 2019 to March 31, 2024) using the same search terms as before. Studies were included if they met the following criteria: (1) studies with ≥10 cases, (2) follow-up duration of ≥6 months, and (3) outcomes reported as favorable (mRS 0–2) and poor (mRS 3–5 or 3–6). Corresponding authors of the included studies were then contacted to request the year of NMDARE diagnosis (or confirmation of whether the diagnosis was before or after 2013), and the last available mRS score for each patient (or, if individual scores were unavailable, whether outcomes was categorized as mRS 0–2 vs. 3–5 or 3–6). Whenever possible, patients were grouped into pre-2013 and post-2013 cohorts based on their year of diagnosis to evaluate the impact of practice changes over time on outcomes. We selected 2013 because it was the year a landmark retrospective cohort analysis in NMDARE was published (Titulaer et al., 2013), substantially influencing current treatment considerations. To minimize duplicate cases, studies were excluded if a larger cohort study was available from the same region or if the study used a nationwide cohort that might overlap with other included studies. Additionally, studies reporting outcome data differently (e.g., median or mean mRS, or dividing mRS into 0–1 or 0–3 as favorable outcome) were Zexcluded from this analysis.

### 2.3. Statistical analysis

Patient demographics, clinical data (including the proportion of intensive care unit [ICU] admissions, mechanical ventilation use, and hospital stays of ≥60 days), investigations, treatments, and functional outcomes were summarized using descriptive statistics. Categorical variables were presented as frequencies (percentages) and compared using the Chi-square test or Fisher’s exact test as appropriate. Continuous variables following a normal distribution were summarized as mean ± standard deviation. Data not following a normal distribution were summarized as median with interquartile range (IQR) and compared using the Mann-Whitney *U* test. Statistical significance was set at *p* < 0.05. Analyses were conducted using R (version 4.3.2; R Foundation).

Multivariable logistic regression models were used to analyze the patient cohorts, stratified by primary outcomes: poor mRS score and relapse. Cohort A focused on poor mRS and included patients with a recorded mRS at last follow-up and ≥ 12 months of follow-up. Patients with an mRS score of 6 (death) with less than 12 months of follow-up were also included in Cohort A. Cohort B focused on relapse. Patients were defined as having relapsed if they were reported to have had a relapse at any time; patients with follow-up of ≥24 months and no relapse at any time were defined as no relapse. Missing values in the resulting datasets were imputed using a hot deck method, where missing data were filled using values from similar cases based on weighted univariate associations with the target outcome (functional outcome at 12 months or relapse). Stability of the imputed values was assessed through sensitivity tests involving ±1 %, ±5 %, and ± 10 % changes in the univariate weights; the final imputed dataset differed from the reference imputation by less than 2 % in all scenarios. The results of these multivariable logistic regression models are reported as odds ratios (OR), 95 % confidence intervals (CI), and *p*-values. Regression models were implemented using the Statsmodels Logit() function in Python 3.8. Sensitivity analyses included bootstrap logistic regression with 10,000 iterations conducted on the full cohorts, providing distributions of regression coefficients to estimate OR, CI, and p-values confirming the robustness of the main results. To ensure model stability, we addressed issues of perfect separation and multicollinearity by removing variables that prevented model convergence and checking variance inflation factors to assess collinearity. In some bootstrap iterations, perfect separation of certain variables occurred, but this did not substantially alter the conclusions. Logistic regression models were chosen as the primary method for models predicting functional outcomes at 12 months and relapse due to their reliability and stability in estimating associations. This held even when the data used had low cell counts in some variables. Bootstrap analysis was kept as a supplementary method to confirm the robustness of results, which aligned with our goal of validating model stability.

## 3. Results

### 3.1. Result of the systematic literature review for individual patient data

**Fig. 1.**
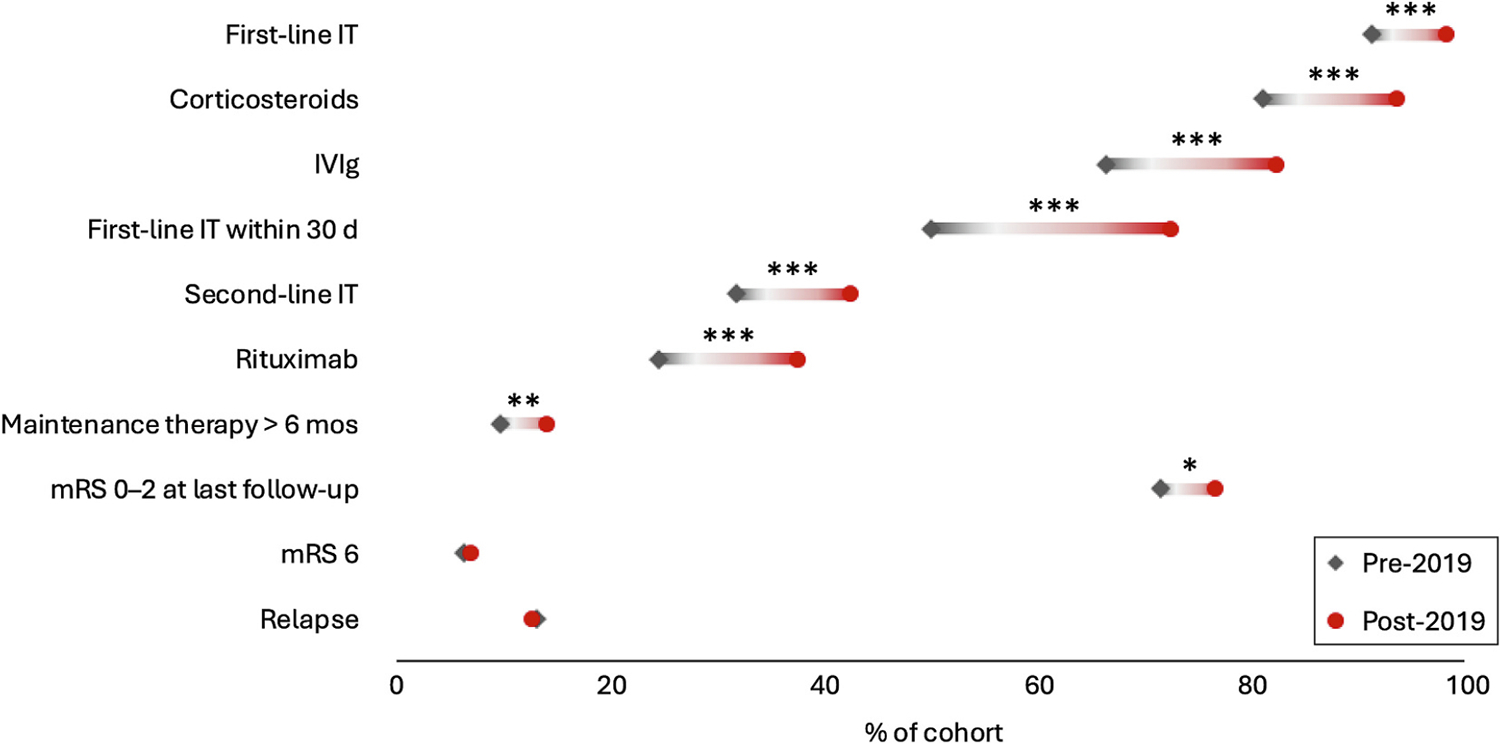
Changes in treatment strategies and outcomes of anti-NMDAR encephalitis d, day; IT, immunotherapy; IVIg, intravenous immunoglobulin; mos, months; mRS, modified Rankin Scale, * *p* < 0.05, ** *p* < 0.005, *** *p* < 0.001.

The literature search retrieved 933 articles published after 2019, of which 321 met the inclusion criteria, representing 649 unique patients from 39 countries (Supplement 1). Of these, 260 papers included only one case each, accounting for 40 % of all cases (Supplement 2). The largest number of patients originated from China (*n* = 194), followed by the United States (*n* = 113), India (*n* = 55), Japan (*n* = 45), and South Korea (*n* = 37) (Supplement 3). Approximately 40 % of the included publications were categorized as describing relatively typical cases of NMDARE. In contrast, the majority of papers (61 %) highlighted atypical or complex aspects of the disease, including unusual clinical presentations (24 %), relapsing or treatment-refractory courses (13 %), unusual demographic / diagnostic testing result (11 %), and emerging / experimental treatment (8 %) or unusual neoplasm association (3 %) (Supplement 4). Demographic characteristics, clinical data, investigations, and disease course in our cohort were compared to the review paper summarizing patients prior to 2019 (Table 1) (Titulaer et al., 2013; Nosadini et al., 2021b). 70.4 % of patients were female. Median age was 21 years with IQR of 14–30 (range 0.3–78 years). The proportion of patients with onset of NMDARE at 18 years or younger was lower than reported in the pre-2019 cohort (40.7 % vs 46.3 %, *p* = 0.017). Tumors were detected in 214/600 patients (35.7 %), with ovarian teratoma or another ovarian tumor in 190/214 patients (88.8 %). The distribution of the mRS scores in the acute phase were similar between the pre-2019 (median [IQR]: 5 [4–5]) and post-2019 (median [IQR]: 5 [4–5]) cohorts, (*p* = 0.99). The rate of ICU admission remained similar (50.6 % vs. 51.5 %, *p* = 0.813), whereas the proportion of patients requiring mechanical ventilation increased (36.2 % vs. 43.2 %, *p* = 0.022) and the proportion of patients requiring hospitalization for ≥60 days decreased (67.0 % vs. 48.0 %, *p* < 0.001). Since 2019, the use of first- (91.3 % vs. 98.3 %, p < 0.001) and second-line (31.8 % vs. 42.5 %, p < 0.001) immunotherapies increased (Table 2, Fig. 1). Notably, increases were seen in the use of corticosteroids (81.1 % vs. 93.7 %, p < 0.001), IVIg (66.4 % vs. 82.4 %, p < 0.001), rituximab (24.5 % vs. 37.6 %, p < 0.001), and bortezomib (1.1 % vs. 4.2 %, p < 0.001). The proportion of patients receiving first-line immunotherapy within 30 days of symptom onset also increased (50.1 vs. 72.5 %, p < 0.001). Additionally, more patients received maintenance immunotherapy for ≥6 months (9.7 % vs. 14.1 %, *p* = 0.004). These trends in the increased use of first-line, second-line, and maintenance immunotherapies, as well as earlier initiation of treatment, were also observed when comparing the post-2019 cohort to the cohort restricted to 2013–2019. Following these therapeutic changes, the post-2019 cohort demonstrated improved functional outcomes compared to the pre-2019 cohort, with a higher proportion of patients achieving an mRS 0–2 score (76.7 % vs. 71.5 %, *p* = 0.024) and 31 % greater odds of achieving an mRS 0–2 (OR 1.31; 95 % CI, 1.04–1.65, *p* = 0.021), while mortality (mRS 6: 6.9 % vs. 6.3 %, *p* = 0.78) and relapse rates (12.6 % vs. 13.2 %, *p* = 0.789) remained similar (Table 3). When comparing the post-2019 cohort to the cohort restricted to 2013–2019 using a chi-square test, the proportion of patients who achieved an mRS 0–2 at last follow-up was not statistically significant (76.6 % vs. 72.0 %, *p* = 0.052). However, the odds of attaining mRS 0–2 were 28 % higher (OR 1.28; 95 % CI, 1.01–1.63; *p* = 0.045). Mortality (mRS 6: 6.9 % vs. 6.6 %, *p* = 0.88) and relapse rates (12.6 % vs. 12.7 %, *p* = 1.00) were comparable in both cohorts.

**Fig. 2.**
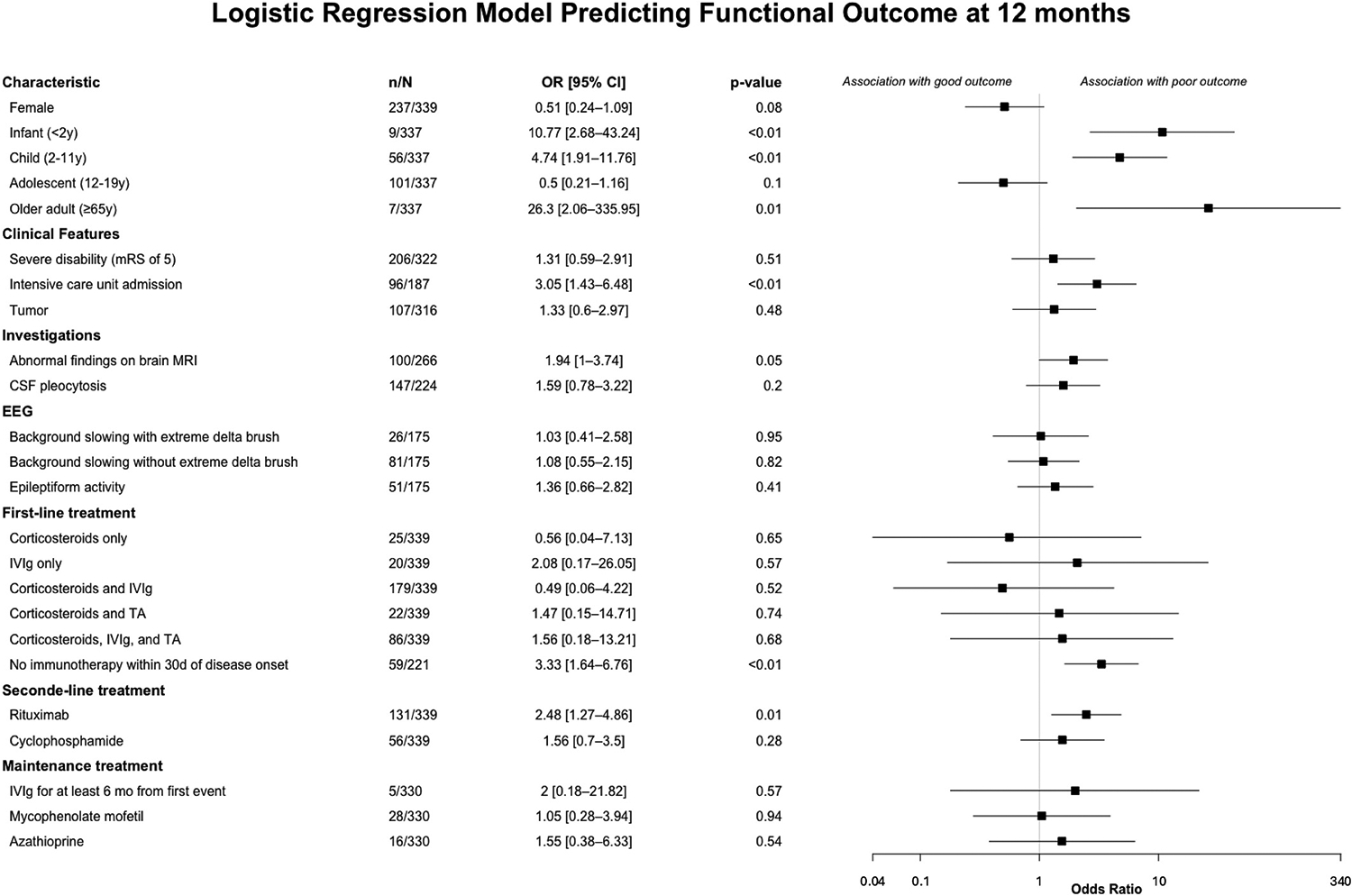
Independent association of clinical characteristics and treatment factors with functional outcome at 12 months. CSF, cerebrospinal fluid; d, day; EEG, electroencephalogram; IVIg, intravenous immunoglobulin; mos, months; MRI, magnetic resonance imaging; mRS, modified Rankin Scale; TA, therapeutic apheresis; y, years.

A total of 339 patients were analyzed as part of the model predicting functional outcome at 12 months with 80 patients (23.6 %) experiencing a poor outcome (mRS score of 3–6). In the logistic regression model (Fig. 2), patient characteristics associated with increased odds of poor outcome were age < 2 years (OR 10.77; 95 % CI 2.68–43.24; *p* < 0.01), age 2–11 years (OR 4.74; 95 % CI 1.91–11.76; p < 0.01), or age ≥ 65y at symptom onset (OR 26.3; 95 % CI 2.06–335.95; *p* = 0.01), and ICU admission (OR 3.05; 95 % CI 1.43–6.48; p < 0.01). Treatment factors associated with poor outcome were lack of immunotherapy within 30 days of symptom onset (OR 3.33; 95 % CI 1.64–6.76; p < 0.01) and use of rituximab as second-line treatment (OR 2.48; 95 % CI 1.27–4.86; p = 0.01). The mRS at nadir in the acute phase was statistically higher among patients who received rituximab compared with those who did not (median [IQR], 5 [5–5] vs. 5 [4–5], *p* = 0.002). No patient characteristics or treatment factors were associated with good outcomes in this analysis. Bootstrap analysis did not reveal any additional factors associated with poor or good outcomes (Supplement 5).

**Fig. 3.**
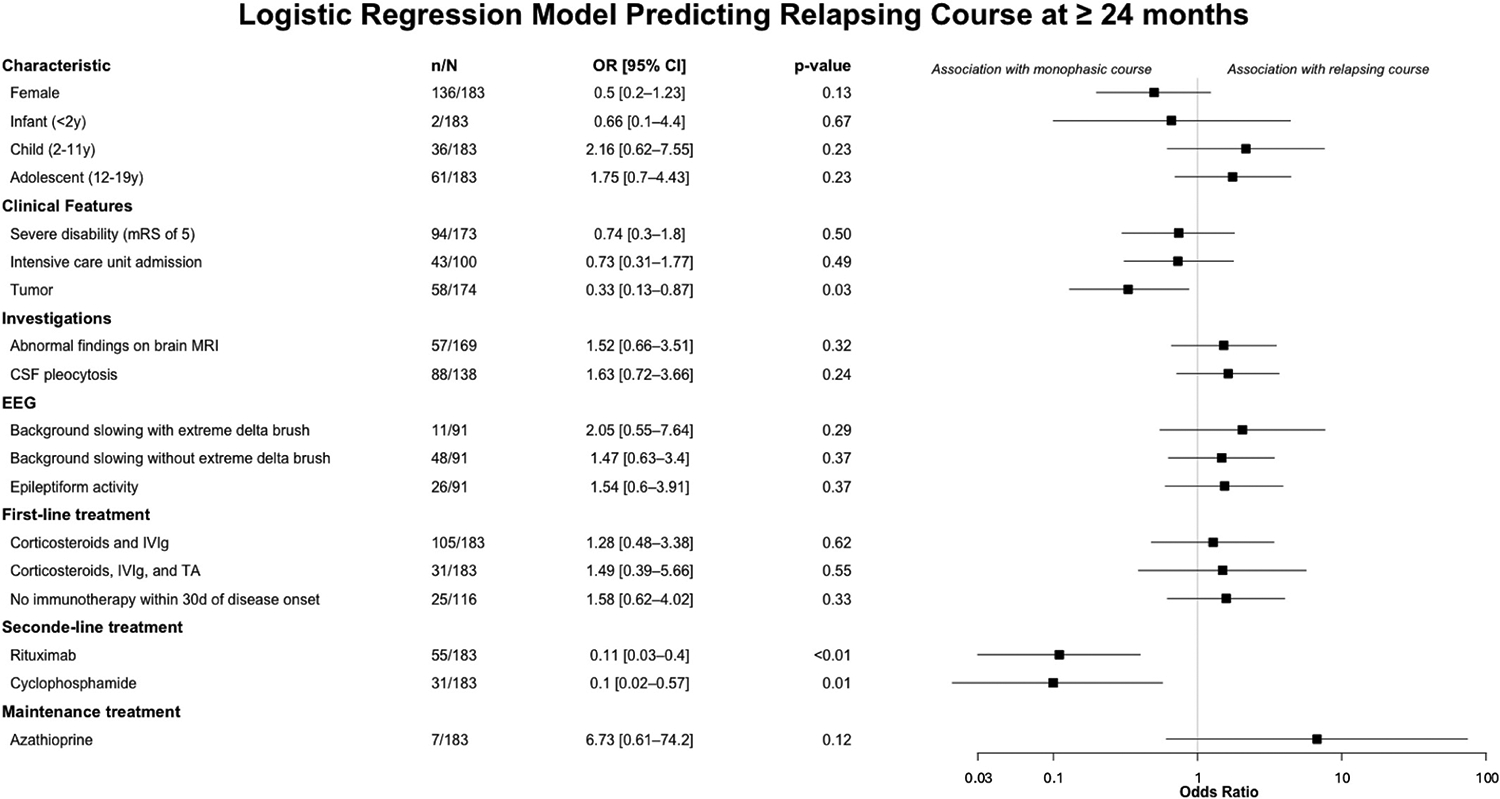
Independent association of clinical characteristics and treatment factors with relapsing disease course at 24 months and later. CSF, cerebrospinal fluid; d, day; EEG, electroencephalogram; IVIg, intravenous immunoglobulin; MRI, magnetic resonance imaging; mRS, modified Rankin Scale; TA, therapeutic apheresis; y, years.

183 patients were analyzed as part of the model predicting relapse at 24 months, including 57 patients (31.1 %) who experienced a relapse during the reported time. In the logistic regression model (Fig. 3), the only patient factor associated with a monophasic disease course was the presence of a tumor at the time of diagnosis (OR 0.33; 95 % CI 0.13–0.87; *p* = 0.03). Treatment factors associated with a monophasic disease course were second-line immunotherapy—specifically use of rituximab (OR 0.11; 95 % CI 0.03–0.4; *p* < 0.01) or cyclophosphamide (OR 0.1; 95 % CI 0.02–0.57; *p* = 0.01). No patient or treatment factors were associated with a relapsing course. Bootstrap analysis used as a supplementary method to confirm the robustness of these results confirmed these and also showed a signal for azathioprine association with a relapsing course (OR 7.16; 95 % CI 1.39–20.20; *p* = 0.02) (Supplement 6).

### 3.2. Result of larger cohort data

Larger cohort studies published after January 2019 reporting favorable (mRS 0–2) and poor (mRS 3–5 or 3–6) outcomes with at least 6 months of follow-up were reviewed and summarized (Supplement 7). In total, 12 such studies were included; nine of them were further categorized by diagnosis period as pre-2013 and post-2013 (Table 4) (Bastiaansen et al., 2022; Lee et al., 2021; Aungsumart et al., 2019; Raja et al., 2021; Guasp et al., 2022; Kong et al., 2019; Xu et al., 2020; Yeshokumar et al., 2022; Morgan et al., 2024; Gombolay et al., 2023; Flet-Berliac et al., 2023; Nikolaus et al., 2023). These studies reported a wide range of favorable outcomes at the last follow-up, from 53 % to 91 % before 2013, and from 56 % to 93 % afterward.

## 4. Discussion

Randomized controlled trials of treatments in patients with NMDARE have yet to be completed and no large, multicenter, prospective treatment studies have been performed. As such, treatment decisions continue to be informed solely by data from retrospective case reports, case series, and cohort studies as well as expert opinions and consensus recommendations. In this comprehensive literature review of publications after January 1, 2019, we reviewed changed in treatment and outcomes, and assessed factors associated with outcomes and relapses and compared them to a prior study (Nosadini et al., 2021b).

Factors that predicted poor outcomes at 12 months in both meta-analyses included age < 2 years or age ≥ 65 years at the time of symptom onset, ICU admission, and no immunotherapy within 30 days of symptom onset (Nosadini et al., 2021b). The current study also identified age 2–11 years at symptom onset and the use of rituximab with poor outcomes — factors which were not identified in the prior meta-analysis (Nosadini et al., 2021b). Several associations were not recapitulated in the updated analysis, including some associated with good outcomes (age 12–19 at symptom onset; first-line immunotherapy with therapeutic apheresis only; first-line immunotherapy with corticosteroids and IVIg; and first-line immunotherapy with corticosteroids, IVIg, and therapeutic apheresis) and some associated with poor outcomes (EEG findings of background slowing with extreme delta brush, the use of IVIg for >6 months after symptom onset) (Nosadini et al., 2021b). These data, when considered along with the data from a large cohort study published in 2013 (Titulaer et al., 2013) and a subsequent study used to identify factors associated with 1-year functional outcomes in NMDARE (Balu et al., 2018), confirm that delayed first-line treatment and ICU admission are poor prognostic factors. Our data suggesting that the use of rituximab is associated with poor outcomes at 12 months was not seen in the prior studies and likely represents selection bias, as patients with poor response to first-line immunotherapy are more likely to receive second-line immunotherapy with agents such as rituximab. Indeed, a secondary analysis showed that patients who received rituximab had a worse mRS in the acute phase than those who did not receive rituximab.

The relapse frequency across the included studies was 12.6 % (Table 3), which is in line with previously reported numbers. However, when comparing the subgroup of patients included in the logistic regression model for factors associated with a relapsing course at ≥24 months (patients who experienced a relapse at any time and those with ≥24 months of follow-up without relapse), our systematic literature review revealed a relapse rate of 31.1 %. This rate is lower than the 44.3 % reported in the prior systematic review (Nosadini et al., 2021b), yet notably higher than the 12 % relapse risk reported in the 2013 cohort study (Titulaer et al., 2013). This discrepancy likely reflects the bias towards publication of dramatic outlier cases rather than an increase in the relapse rate over time or a population bias based on the higher number of publications from China than any other region. The only factor that was associated with a monophasic disease course in the current and prior literature review was the use of rituximab as second-line immunotherapy. (Titulaer et al., 2013; Nosadini et al., 2021b) In the current study, the presence of a tumor at the time of diagnosis or the use of cyclophosphamide were also associated with a monophasic disease course. A large cohort study published in 2013 showed that patients without a tumor had a higher frequency of relapses (*p* = 0.0007) (Titulaer et al., 2013), and the previous literature review suggested that tumor may associate with a monophasic disease course (*p* = 0.06) (Nosadini et al., 2021b). Prospective studies of patients with NMDARE may help clarify whether the presence of a tumor is an independent predictor for a monophasic disease course. The previous literature review also showed that the use of IVIg for >6 months was associated with a monophasic disease course based on a small subset of patients, while a relapsing disease course was associated with age 12–19 at symptom onset—factors which were not demonstrated in the current study (Nosadini et al., 2021b). A secondary analysis of our data suggested that the use of azathioprine was associated with a relapsing course, though this likely represents treatment bias as patients with relapses may have been more likely to be started on a maintenance immunotherapy such as azathioprine.

The findings of the current report as well as an earlier publication which used similar methodology highlight some of the weaknesses with such studies. Specifically, case reports, case series, and cohort studies offer Class III and Class IV levels of evidence (Gross and Johnston, 2009) which are subject to reporting and publication bias, confirmation bias from unmasked and unblinded assessments, and selection bias; such bias may compromise reproducibility between studies. In rare diseases, authors are more likely to publish cases with either very good or very bad outcomes, limiting the applicability of any subsequent pooled analysis of data to the broader population of patients with the rare disease due to this reporting bias. In fact, 40 % of the included cases came from single-patient case reports, and 75 % were included from publications with 10 or fewer cases (Supplement 2). Patients included from 126 publications (39 %) had relatively typical NMDARE clinical presentations and treatment courses, whereas the remaining 195 publications (61 %) emphasized atypical features or novel aspects—including experimental treatments and/or diagnostic technologies—with the most common being unusual clinical presentations (Supplement 4). This highlights that published reports may disproportionately represent atypical cases, reinforcing the need for prospective, registry-based studies to obtain more accurate and unbiased estimates of treatment response and long-term outcomes. Confirmation bias may result in higher numbers of cases with pathognomonic findings being reported, such as the 31.5 % rate of ovarian teratoma seen in the current study, while the prior study showed a rate of only 20.5 %. The idiosyncrasies of individual or regional clinical practice may also contribute to selection bias, making it challenging to pool data in a meta-analysis meaningfully (Twine and Mani, 2022). We have made efforts to highlight some of these biases in the discussion above, and any conclusions the reader may draw from the current study must keep these in mind.

In addition to bias, statistical models may show substantial differences between certain factors, but when these factors represent a very small number of cases which may be outliers, a significant *p* value is not necessarily clinically relevant and may not be reproducible in subsequent studies. In addition, statistical trends must be considered within the appropriate clinical context and not interpreted in isolation.

The current study as well as previous studies demonstrate that the majority of patients improve with first-line immunotherapy alone, and yet nearly 40 % of patients receive second-line immunotherapy despite no evidence that this improves outcomes for all patients (Supplement 8). Our review of the literature as well as previous studies suggest that the use of rituximab is associated with a monophasic disease course, but this evidence is not strong enough to suggest that all patients should receive a B-cell depleting agent as standard of care in NMDARE. There is no clinical trial data to support the use of rituximab in NMDARE, yet it is increasingly being used immediately after the completion of first-line immunotherapy. Although generally well-tolerated, some patients have severe adverse events including life-threatening infections and infusion reactions (Kasi et al., 2012; McAtee et al., 2021). There is equipoise for clinical trials evaluating which patients need rituximab (or any second-line immunotherapy) as well as the appropriate timing of treatment initiation and duration of use.

Practice patterns in NMDARE have continued to evolve along with published data and clinical experience. Since 2019, the use of first- and second-line immunotherapy in published reports, as well as earlier initiation of immunotherapy, has increased. Off-label second-line/maintenance immunotherapy use has also become more common. These trends were seen when comparing the post-2019 cohort with both the pre-2019 cohort and a cohort restricted to 2013–2019. However, despite these changes, the proportion of patients achieving good outcomes (mRS 0–2) at last follow-up improved only modestly with no statistically significant change in relapse rates or mortality. The reasons for increased use of immunotherapy over time cannot be inferred from the current or prior studies, but may reflect increasing recognition of NMDARE, availability of published treatment consensus recommendations, or publication bias rather than an actual practice change. The apparent disconnect between treatments and outcomes emphasizes the need for higher quality data to inform treatment decisions. Furthermore, outcome measurements based on mRS insufficiently address persistent symptoms and disability in patients with NMDARE including cognitive deficits, fatigue, and impaired social functioning (Heine et al., 2021; Brenner et al., 2024; Heine et al., 2024). Large, collaborative, prospective studies and clinical trials are well-suited to address issues related to the appropriate timing and use of second-line immunotherapy and to incorporate more nuanced outcome measures. Unfortunately, the logistics of conducting prospective studies often limit participation to patients able to reach a few large academic centers in the world, and while clinical trials may provide the infrastructure and incentives to broaden patient access, enrollment in such trials has been challenging (Abboud et al., 2025).

This study has several limitations. Its retrospective design makes it vulnerable to selection, publication, confirmation, and recall biases. With 81 % of publications as single case reports and nearly half of the cases coming from China and the U.S., regional practice patterns or ethnic differences in disease expression may limit generalizability to a broader population. Outcome measures were limited to mRS, which fails to capture many reported long-term deficits in patients with NMDARE. These limitations require well-designed prospective studies to be addressed.

## 5. Conclusion

Since 2019, first- and second-line off-label immunotherapies have been used more frequently to treat patients with NMDARE, and the proportion of patients receiving first-line therapy within 30 days of symptoms onset has increased. Likewise, off-label second-line/maintenance immunotherapy use has become more common. These changes occur concurrent with modest improvements in functional outcomes, without changes in mortality and relapse rates. Overall, there remains ongoing clinical equipoise, and an urgent need for robust, prospective studies to determine the most effective treatment strategies in patients with NMDARE.

## Supplementary Material

Suplementary Material

Supplementary data to this article can be found online at https://doi.org/10.1016/j.jneuroim.2025.578651.

## Figures and Tables

**Table 1:** Demographic characteristics, clinical data, investigations, and disease course in the cohort of patients with anti-NMDAR encephalitis with individual patient treatment data available.

	Before 2019^9^ *N=* 1550 (%)	2013 to 2019^9^ *N=* 1163 (%)	After 2019 *N=* 649 (%)	P value^a^	
				Before vs After 2019	2013–2019 vs After 2019
Total number of articles	652	424	321	NA	NA
**DEMOGRAPHICS**					
Total number of patients	1550	1163	649	NA	NA
Proportion of females	1105/1508 (73.3)	795/1121 (70.9)	457/649 (70.4)	0.19	0.87
Proportion ≤ 18 years	707/1526 (46.3)	546/1139 (47.9)	264/649 (40.7)	0.02	0.004
Age at onset of NMDARE (years)	Median 20, mean 23, range 0–85 (d.a.:1517/1550)	Median 19, mean 23.1, range 0–85 (d.a.:1131/1163)	Median 21, mean 23, range 0.3–78 (d.a.:645/649)	NA	NA
**CLINICAL DATA AT FIRST EVENT OF NMDAR**					
Days from symptom onset to hospitalization	Median 10, mean 30.3, range 0–2190 (d.a.:452/1163)	Median 10, mean 35.1, range 0–2190 (d.a.:310/1163)	Median 10, mean 31.8, range 0–1095 (d.a.:352/649)	NA	NA
**mRS nadir in the acute phase**					
mRS 1	0/1113 (0)	0/793 (0)	1/599 (0.2)	0.99^b^	0.34^b^
mRS 2	8/1113 (0.7)	8/793 (1)	10/599 (1.7)		
mRS 3	144/1113 (12.9)	117/793 (14.8)	75/599 (12.5)		
mRS 4	309/1113 (27.8)	220/793 (27.7)	159/599 (26.5)		
mRS 5	652/1113 (58.6)	448/793 (56.5)	354/599 (59.1)		
**Admission to the intensive care unit**	488/964 (50.6)	323/664 (48.6)	228/443 (51.5)	0.81	0.39
**Mechanical ventilation**	302/834 (36.2)	198/566 (35)	171/396 (43.2 %)	0.02	0.01
**Length of hospitalization ≥ 60 days**	225/336 (67)	125/210 (59.5)	156/325 (48)	<0.001	0.01
**Associated tumor**	389/1524 (25.6)	257/1147 (17.3)	214/600 (35.7)	<0.001	**<**0.001
Ovarian teratoma	312/1524 (20.5)	198/1147 (17.3)	189/600 (31.5)	<0.001	**<**0.001
Other ovarian tumor	12/1524 (0.8)	5/1147 (0.4)	1/600 (0.2)	0.13	0.67
Other tumor (extra-ovarian tumor)	66/1524 (4.3)	54/1147 (4.7)	24/600 (4)	0.82	0.58
**INVESTIGATIONS AT FIRST EVENT OF NMDARE**					
**Abnormal brain MRI**	434/1069 (40.6)	275/741 (37.1)	195/543 (35.9)	0.08	0.70
**Abnormal EEG**	725/855 (84.8)	481/575 (83.7)	372/466 (79.8)^c^	0.03	0.13
Focal or diffuse slow disorganized activity	594/822 (72.3)	385/542 (71)	223/423 (52.7)	**<**0.001	**<**0.001
Epileptic activity (epileptic discharges)	244/829 (29.4)	149/548 (27.2)	64/423 (15.1)	**<**0.001	**<**0.001
Seizures recorded	132/829 (15.9)	67/548 (12.2)	49/423 (11.6)	0.05	0.84
Status epilepticus recorded	43/827 (5.2)	19/547 (3.5)	23/423 (5.4)	0.96	0.18
Extreme delta brush	56/827 (6.8)	55/547 (10.1)	64/423 (15.1)	**<**0.001	0.02
Periodic lateralized epileptic discharges (PLEDs)	13/827 (1.6)	6/547 (1.1)	1/423 (0.2)	0.04	0.15
**Abnormal CSF**					
Pleocytosis >4 cells/uL	618/911 (67.8)	393/615 (63.9)	287/424 (67.7)	1	0.23
Hyperproteinorrachia >50 mg/dL	189/873 (21.6)	134/580 (23.1)	101/387 (26.1)	0.1	0.32
Positive oligoclonal bands	223/356 (62.6)	124/213 (58.2)	53/94 (56.4)	0.33	0.86
**NMDAR-antibodies**					
Positive in serum	702/796 (88.2)	496/570 (87)	234/288 (81.3)	0.005	0.03
Positive in CSF	973/986 (98.7)	721/731 (98.6)	635/635 (100)	0.003	0.002

CSF, cerebrospinal fluid; d.a., data available; EEG, electroencephalography; MRI, magnetic resonance imaging; mRS, modified Rankin Scale; NMDARE, NMDAR-antibody encephalitis.

a Fisher’s Exact Test was applied to variables with counts less than 5 to ensure statistical accuracy, while the Chi-Square Test was used for counts of 5 or greater to assess the independence of the variables.

b Mann-Whitney *U* test used to compare the mRS distributions between the different time periods.

c 45 patients had other EEG abnormalities: 1 patient had “extreme spindles,” 1 patient had “high frequency oscillations,” and 43 patients had “abnormal” finding (detail unspecified).

**Table 2:** Descriptive data on immunotherapeutic management in the cohort of patients with anti-NMDAR encephalitis with individual patient treatment data available.

TREATMENT	Before 2019^9^ N = 1550 (%)	2013 to 2019^9^ N = 1163 (%)	After 2019 N = 649 (%)	P value^a^	
				Before 2019 vs After 2019	2013–2019 vs After 2019
**First-line immunotherapy**	1395/1528 (91.3)	1057/1142 (92.6)	638/649 (98.3)	**<**0.001	**<**0.001
Corticosteroids	1205/1485 (81.1)	907/1100 (82.5)	608/649 (93.7)	**<**0.001	**<**0.001
IVIg	980/1476 (66.4)	748/1091 (68.6)	535/649 (82.4)	**<**0.001	**<**0.001
TA	500/1482 (33.7)	376/1097 (34.3)	226/649 (34.8)	0.66	0.86
**Second-line immunotherapy**	486/1526 (31.8)	406/1142 (35.6)	276/649 (42.5)	**<**0.001	0.004
Rituximab	363/1484 (24.5)	311/1100 (28.3)	244/649 (37.6)	**<**0.001	**<**0.001
Cyclophosphamide	184/1484 (12.4)	142/1100 (12.9)	90/649 (13.9)	0.39	0.62
Bortezomib	16/1484 (1.1)	16/1100 (1.5)	27/649 (4.2)	**<**0.001	**<**0.001
Tocilizumab	11/1484 (0.7)	11/1100 (1)	11/649 (1.7)	0.08	0.3
Intravenous or intrathecal methotrexate	10/1484 (0.7)	10/1100 (0.9)	2/649 (0.3)	0.37	0.23
Other	4/1484 (0.3)	4/1100 (0.4)	5/649 (0.8)	0.14	0.31
**Maintenance immunotherapy ≥ 6 mo**	146/1508 (9.7)	114/1123 (10.2)	87/615 (14.1)	0.004	0.02
Mycophenolate mofetil	43/1508 (2.9)	38/1142 (3.3)	37/615 (6)	**<**0.001	0.01
Azathioprine	39/1508 (2.6)	27/1143 (2.4)	25/615 (4.1)	0.1	0.06
IVIG	24/1508 (1.6)	18/1140 (1.6)	6/615 (1)	0.38	0.41
Methotrexate	17/1508 (1.1)	17/1145 (1.5)	6/615 (1)	0.94	0.5
Corticosteroids	40/1508 (2.7)	29/1145 (2.5)	4/615 (0.7)	0.002	0.005
Rituximab redosing	7/1508 (0.5)	6/1143 (0.5)	13/615 (2.1)	**<**0.001	0.005
Other	4/1508 (0.3)	3/1140 (0.3)	5/615 (0.8)	0.16	0.14
**Time to first immunotherapy ≤ 30 d**	365/728 (50.1)	268/550 (48.7)	306/422 (72.5)	**<**0.001	**<**0.001
**Overall immunotherapy combinations**					
First-line immunotherapy only	826/1526 (54.1)	590/1142 (51.7)	327/649 (50.4)	0.12	0.64
First-line + second-line immunotherapy only	421/1526 (27.6)	353/1142 (30.9)	224/649 (34.5)	0.001	0.13
First-line + second-line + maintenance immunotherapy	60/1526 (3.9)	49/1142 (4.3)	49/649 (7.6)	**<**0.001	0.005
First-line + maintenance immunotherapy only	86/1526 (5.6)	65/1142 (5.7)	38/649 (5.9)	0.92	0.97
Second-line immunotherapy only	5/1526 (0.3)	4/1142 (0.4)	3/649 (0.5)	0.70	0.71
No immunotherapy	128/1526 (8.4)	81/1142 (7.1)	8/649 (1.2)	**<**0.001	**<**0.001
**First-line immunotherapy combination**					
Corticosteroids + IVIG	561/1478 (38)	435/1093 (39.8)	326/649 (50.2)	**<**0.001	**<**0.001
Corticosteroids + IVIG + TA	301/1478 (20.4)	228/1093 (20.9)	180/649 (27.7)	**<**0.001	0.001
Corticosteroids only	199/1478 (13.5)	132/1093 (12.1)	59/649 (9)	0.006	0.06
Corticosteroids + TA	142/1478 (9.6)	110/1093 (10.1)	43/649 (6.6)	0.030	0.02
IVIG only	86/1478 (5.8)	66/1093 (6)	27/649 (4.2)	0.14	0.12
IVIG + TA	35/1478 (2.4)	22/1093 (2)	2/649 (0.3)	**<**0.001	0.002
TA only	22/1478 (1.5)	16/1093 (1.5)	1/649 (0.2)	0.005	0.005
No first-line immunotherapy	132/1478 (8.9)	84/1093 (7.7)	11/649 (1.7)	**<**0.001	**<**0.001
**Second-line immunotherapy combination**					
Rituximab only	244/1484 (16.4)	206/1100 (18.7)	162/649 (25.0)	**<**0.001	**<**0.001
Rituximab + cyclophosphamide	92/1484 (6.2)	78/1100 (7.1)	46/649 (7.1)	0.50	1
Cyclophosphamide only	79/1484 (5.3)	51/1100 (4.6)	30/649 (4.6)	0.57	0.87
Other	30/1484 (2)	30/1100 (2.7)	38/649 (5.9)	**<**0.001	**<**0.001
No second-line immunotherapy	1039/1484 (70)	735/1100 (66.8)	373/649 (57.5)	**<**0.001	**<**0.001

IVIg: Intravenous immunoglobulin; TA: Therapeutic apheresis

a Fisher’s Exact Test was applied to variables with counts less than 5 to ensure statistical accuracy, while the Chi-Square Test was used for counts of 5 or greater to assess the independence of the variables.

**Table 3:** Functional outcome (measured by mRS) data in the cohort of patients with anti-NMDAR encephalitis with individual patient treatment data available.

OUTCOME	Before 2019^9^ N = 1550 (%)	2013 to 2019^9^ N = 1163 (%)	After 2019 N = 649 (%)	P value^a^	
				Before vs After 2019	2013–2019 vs After 2019
**Length of follow-up, mo**					
Patients	1059/1550 (68.3)	780/1163 (67.1)	509/649 (78.4)	NA	NA
Median	12	12	12	NA	NA
Mean	20.7	20.2	20.1		
Range	0.5–268	0.5–250	0.5–180		
Proportion with reported relapse	182/1380 (13.2)	129/1017 (12.7)	57/454 (12.6)	0.79	1
**mRS score at last follow-up**					
Patients	1284/1550 (82.8)	946/1163 (81.3)	566/649 (87.2)	NA	NA
Median	2	2	1	NA	NA
Mean	1.8	1.8	1.4		
Range	0–6	0–6	0–6		
0 to 2	918/1284 (71.5)	681/946 (72)	434/566 (76.7)	0.02	0.052
3 to 5	285/1284 (22.2)	203/946 (21.5)	93/566 (16.4)	0.006	0.02
6	81/1284 (6.3)	62/946 (6.6)	39/566 (6.9)	0.71	0.88

mRS: modified Rankin scale

a Fisher’s Exact Test was applied to variables with counts less than 5 to ensure statistical accuracy, while the Chi-Square Test was used for counts of 5 or greater to assess the independence of the variables.

**Table 4:** Cohort studies (≥10 cases) published during the study period with at least 6 months of follow-up, reporting outcomes as favorable (mRS 0–2) and poor (mRS 3–5 or 3–6), grouped by diagnosis period as pre-2013 and post-2013.

Authors	Patient age		Institution	Country/Area	Time	Total number of ≥ 6 months follow-up		Favorable outcome (mRS 0–2)		Follow-up
	Pre 1/2013	Post 1/2013				Pre 1/2013	Post 1/2013	Pre 1/2013	Post1/2013	
Bastiaansen AEM et al. (Bastiaansen et al., 2022)	Median 22 yrs. (IQR 17–29, Range 4–67)	Median 25 yrs. (IQR 17–40, Range 1–86)	Erasmus University Medical Center	Netherlands	3/2007–12/2019	28	92	15 (53%)	57 (62%)	6 mos
						27	84	20 (74 %)	66 (79%)	12 mos
						25	63	22 (88%)	49 (78%)	24 mos
Lee WJ et al. (Lee et al., 2021)	N.A.	Mean ± SD is 27.4 ± 13.4 yrs. (Range: 6–78)	Seoul National University Hospital	Korea	1/2014–10/2019	N.A.	78	N.A.	58 (74%)	Mean 23.2 mos (Range: 6–24 mos)
Aungsumart S et al. (Aungsumart et al., 2019)	Median 17.0 yrs. (IQR 15–31.5)	Median 23.5 yrs. (IQR 15.8–29.5)	Prasat Neurological Institute	Thailand	1/2011–12/2016	19	16	14 (74%)	9 (56 %)	12 mos
Raja P et al. (Raja et al., 2021)	N.A.	Median 14 yrs. (Range 2–61)	National Institute of Mental Health and Neurosciences	India	1/2018–3/2020	N.A.	28	N.A.	19 (68%)	12 mos
							19		16 (84%)	24 mos
Guasp et al. (Guasp et al., 2022)	N.A.	Median 27 yrs. (IQR 21–35, Range 13–57)	Hospital Clínic de Barcelona	Spain	1/2017–9/2020	N.A.	26	N.A.	22 (85%)	Median 16 mos (IQR 15–19)
Kong SS et al. (Kong et al., 2019)	Mean ± SD is 22.7 ± 7.6 yrs	Mean ± SD is 15.8 ± 7.1 yrs	Chang Gung Children’s Hospital	Thaiwan	2009–2017	3	21	2 (67 %)	18 (86%)	6 mos
Xu X et al. (Xu et al., 2020)	Median 21 yrs. (Range 5–72)		Peking Union Medical College Hospital	China	5/2011–12/2017	220		204 (9	3 %)	12 mos
Yeshokumar A et al. (Yeshokumar et al., 2022)	Mean ± SD is 19.1 ± 16.9 yrs		Johns Hopkins Hospital, Hospital of the University of Pennsylvania, and Children’s Hospital of Philadelphia	U.S.	7/2005–6/2015	40		35 (88	%)	Mean ± SD is 4 ± 2.4 yrs
Morgan A et al. (Morgan et al., 2024)	Median 28 yrs.	(IQR 22–36, Range 1–75)	Cleveland Clinic	U.S.	N/A	31		22 (71	%)	Median 70 weeks (IQR 51–174)
**Pediatric-only cohort**										
Gombolay G et al. (Gombolay et al., 2023)	Mean ± SD is 11.0 ± 4.8 yrs	Mean ± SD is 10.6 ± 5.2 yrs	11 institutions in the U.S.	U.S.	1/2008–9/2022	34	151	27 (79%)	132 (87%)	12 mos
						11	60	10 (91%)	56 (93%)	24 mos
Flet-Berliac L et al. (Flet-Berliac et al., 2023)	Median 10.7 yrs. (IQR 5.2–14.2, Range 1.1–16.7)	Median 10.3 yrs. (IQR 6.3–15.5, Range 1.1–17.8)	18 tertiary pediatric neurology centres in France	France	12/2007–9/2015	34	21	30 (88%)	19 (90%)	24 mos
						36	38	32 (89%)	34 (90%)	Last follow-up (Median 42 mos, Range 8–121)
Nikolaus M et al. (Nikolaus et al., 2023)	N.A.	Median 8 yrs. (Range 0.8–17)	12 sites in Europe (Germany, Austria, and Sweden)	Europe	2020–2021	N.A.	56	N.A.	49 (88%)	Median 20 mos (Range: 12–52 mos)

IQR, Interquartile range; mos, months; mRS, modified Rankin scale; N.A., no answer; yrs., years.

## Data Availability

The datasets generated for this study are available upon reasonable request. The raw data from the systematic literature review supporting the conclusions of this article will be made available by the author. Requests to access the datasets should be directed to Y.H., Yoji.Hoshina@hsc.utah.edu. Please note that the large cohort datasets (from the five separate cohorts obtained independently) must be requested from the respective corresponding authors of the original publications.
