# Supplementary material for: An updated, comprehensive meta-analysis of the treatment of anti-NMDAR encephalitis: Analysis, equipoise, and the urgent need for evidence over anecdote
Source: J Neuroimmunol. Author manuscript; Available in PMC 2026 Aug 15. (PMC13295269; doi:10.1016/j.jneuroim.2025.578651)
Supplement: Supplementary file 1 [file NIHMS2180758-supplement-Suplementary_Material.docx]

**Supplement 1:** PRISMA flow diagram

NMDAR, N-methyl-D-aspartate receptor; NMDARE, Anti-N-methyl-D-aspartate receptor encephalitis

**Supplement 2:** Number of patients extracted per published report

A total of 649 cases were extracted from 321 primary reports. Of these, 260 papers included only one case each, collectively accounting for 40% of all cases. Studies contributing 20 or more cases are indicated as follows: *Tan YJ et al., 2023^31^ ; **Zhang H et al., 2022^32^ ; ***Zhang Y et al., 2019^33^

**Supplement 3:** Geographical distribution of the anti-NMDAR encephalitis cohort (n=649) from 321 included studies

The map displays the number of patients (n=649) from each of the 39 countries represented in the 321 included studies. Countries are colored according to their patient count, with dark red indicating higher numbers and light pink indicating lower numbers. China had the largest cohort (n=194), followed by the United States (n=113), India (n=55), Japan (n=45), and South Korea (n=37).

**Supplement 4:** Categorization of publications describing patient with anti-NMDAR encephalitis including in the study.

| **Category** | **Number of papers** | **% of total** |
| --- | --- | --- |
| Relapsing / treatment-refractory course | 40 | 12.5 % |
| Unusual neoplasm association | 11 | 3.4 % |
| Emerging / experimental treatment | 27 | 8.4 % |
| Emerging diagnostic technology | 5 | 1.6 % |
| Unusual clinical presentation | 78 | 24.3 % |
| Unusual demographic / diagnostic testing result^*^ | 34 | 10.6 % |
| Relatively typical case | 126 | 39.3 % |
| Total | 321 | 100 % |

*Examples include pregnancy, age < 2 y or ≥65 y, or atypical MRI/EEG patterns.

**Supplement 5:** Results from the bootstrapped logistic regression models describing independent association of clinical characteristics and treatment factors with functional outcome at 12 months

CSF, cerebrospinal fluid; d, day; EEG, electroencephalogram; IVIg, intravenous immunoglobulin; mos, months; MRI, magnetic resonance imaging; mRS, modified Rankin Scale; TA, therapeutic apheresis; y, years

**Supplement 6:** Results from the bootstrapped logistic regression models independent association of clinical characteristics and treatment factors with relapsing disease course at 24 months and later

CSF, cerebrospinal fluid; d, day; EEG, electroencephalogram; IVIg, intravenous immunoglobulin; MRI, magnetic resonance imaging; mRS, modified Rankin Scale; TA, therapeutic apheresis; y, years

**Supplement 7:** Diagram of larger cohort studies published after January 2019 reporting favorable (mRS 0–2) and poor (mRS 3–5 or 3–6) outcomes with at least 6 months of follow-up

NMDARE, Anti-N-methyl-D-aspartate receptor encephalitis

**Supplement 8:** Number of patients included in the logistic regression model predicting functional outcome, their receipt of immunotherapy, and their functional outcome at the last follow-up

IT, immunotherapy; mRS, modified Rankin Scale
